# Three New Alpha1-Antitrypsin Deficiency Variants Help to Define a C-Terminal Region Regulating Conformational Change and Polymerization

**DOI:** 10.1371/journal.pone.0038405

**Published:** 2012-06-18

**Authors:** Anna M. Fra, Bibek Gooptu, Ilaria Ferrarotti, Elena Miranda, Roberta Scabini, Riccardo Ronzoni, Federica Benini, Luciano Corda, Daniela Medicina, Maurizio Luisetti, Luisa Schiaffonati

**Affiliations:** 1 Department of Biomedical Sciences and Biotechnology, University of Brescia, Brescia, Italy; 2 Institute of Structural Molecular Biology/Crystallography, Birkbeck College, University of London, London, United Kingdom; 3 Department of Molecular Medicine, University of Pavia, Istituto Di Ricovero e Cura a Carattere Scientifico Policlinico San Matteo, Pavia, Italy; 4 Department of Biology and Biotechnology ‘Charles Darwin’ and Pasteur Institute, Cenci Bolognetti Foundation, University of Rome ‘La Sapienza’, Rome, Italy; 5 Division of Internal Medicine, Spedali Civili, Brescia, Italy; 6 Department of Pathology, Spedali Civili, Brescia, Italy; Consejo Superior de Investigaciones Cientificas, Spain

## Abstract

Alpha1-antitrypsin (AAT) deficiency is a hereditary disorder associated with reduced AAT plasma levels, predisposing adults to pulmonary emphysema. The most common genetic AAT variants found in patients are the mildly deficient S and the severely deficient Z alleles, but several other pathogenic rare alleles have been reported. While the plasma AAT deficiency is a common trait of the disease, only a few AAT variants, including the prototypic Z AAT and some rare variants, form cytotoxic polymers in the endoplasmic reticulum of hepatocytes and predispose to liver disease. Here we report the identification of three new rare AAT variants associated to reduced plasma levels and characterize their molecular behaviour in cellular models. The variants, called Mpisa (Lys259Ile), Etaurisano (Lys368Glu) and Yorzinuovi (Pro391His), showed reduced secretion compared to control M AAT, and accumulated to different extents in the cells as ordered polymeric structures resembling those formed by the Z variant. Structural analysis of the mutations showed that they may facilitate polymerization both by loosening ‘latch’ interactions constraining the AAT reactive loop and through effects on core packing. In conclusion, the new AAT deficiency variants, besides increasing the risk of lung disease, may predispose to liver disease, particularly if associated with the common Z variant. The new mutations cluster structurally, thus defining a region of the AAT molecule critical for regulating its conformational state.

## Introduction

Alpha1-antitrypsin deficiency (AATD) is a genetic disorder characterized by reduced plasma levels of alpha1-antitrypsin (AAT), the archetypal member of the serpin (serine protease inhibitor) protein superfamily. An acute phase glycoprotein, AAT is mainly synthesized in the liver and it inhibits neutrophil elastase to regulate inflammatory processes in the circulation and lung tissue [1], [2]. Plasma levels of AAT are tightly linked to variants of the coding gene *SERPINA1* (14q32.1) (OMIM: 107400), which dictate the AAT concentration according to a co-dominant model. Pathogenic alleles of the *SERPINA1* gene are conventionally classified as “null”, if associated with undetectable AAT, or as “deficient” alleles if the mutated protein is synthesised and retained in a misfolded state within hepatocytes, resulting in variable degrees of secretion defect [3].

The commonest deficient variants causing AATD are the S (Glu264Val) and Z (Glu342Lys) mutations. SS genotypes result in mild deficiency, with minimal clinical sequelae. The ZZ and SZ genotypes, associated with 15% and 25% of normal AAT plasma levels, account for the vast majority of severe AATD. However, there are several other pathogenic AAT alleles described, usually referred to as “rare alleles”, associated with varying degrees of reduced plasma level. The prevalence of these rare AATD genotypes is believed to be more frequent in the Mediterranean area [4], [5].

The deficiency of circulating AAT is associated with an increased risk of developing emphysema, due to uncontrolled elastase activity in the lung, which may be exacerbated by additional risk factors such as cigarette smoke [6]. Besides emphysema, the Z AAT variant also predisposes to liver diseases, which may present in ZZ homozygous individuals as neonatal jaundice, cirrhosis and increased risk of hepatocellular carcinoma [7]. The development of liver disease is caused by excessive accumulation of Z AAT in the endoplasmic reticulum (ER) of hepatocytes as ordered polymeric structures, which deposit in PAS+, diastase-resistant inclusion bodies [8]. Polymerization of Z AAT proceeds through formation of an unstable monomeric AAT intermediate termed M* [9], [10]. This permits a stabilising interaction between two AAT motifs, the reactive loop and beta-sheet A. The latter is opened in M* and insertion of the reactive loop links two AAT molecules into a dimer. Successive intermolecular interactions lengthen this into a polymer chain [11]. Currently three models of serpin polymerization are proposed in the literature, all of which conform to these constraints, but differing in the number of beta strands (1, 2, or 3) involved in the intermolecular linkage [12]–[15]. However all three models incorporate release of strand 1 of beta-sheet C, expansion of beta-sheet A and insertion of reactive loop residues as an extra strand in beta-sheet A. Besides the archetypal Z AAT, other deficiency variants have been shown to form polymers within the ER. While the Z mutation resides in the hinge region at the base of the reactive loop, other strongly polymerizing variants such as Siiyama (Ser52Phe), Mmalton (Phe52del) and King’s (His334Asp) carry mutations localized in the shutter region, which directly favour opening of beta-sheet A [16]–[18]. The Pbrescia mutation instead perturbs an arginine pocket that normally stabilizes the reactive loop [19], and milder polymerizing variants such as I (Arg39Cys) and S (Glu264Val) may act indirectly on beta-sheet A stability by perturbing helix A/G-core packing [20]. Although the different mutations affect different AAT regions, the resulting polymers are recognised by a monoclonal antibody (mAb) specific for Z AAT polymers, strongly supporting their structural similarity [18].

**Figure 1:**
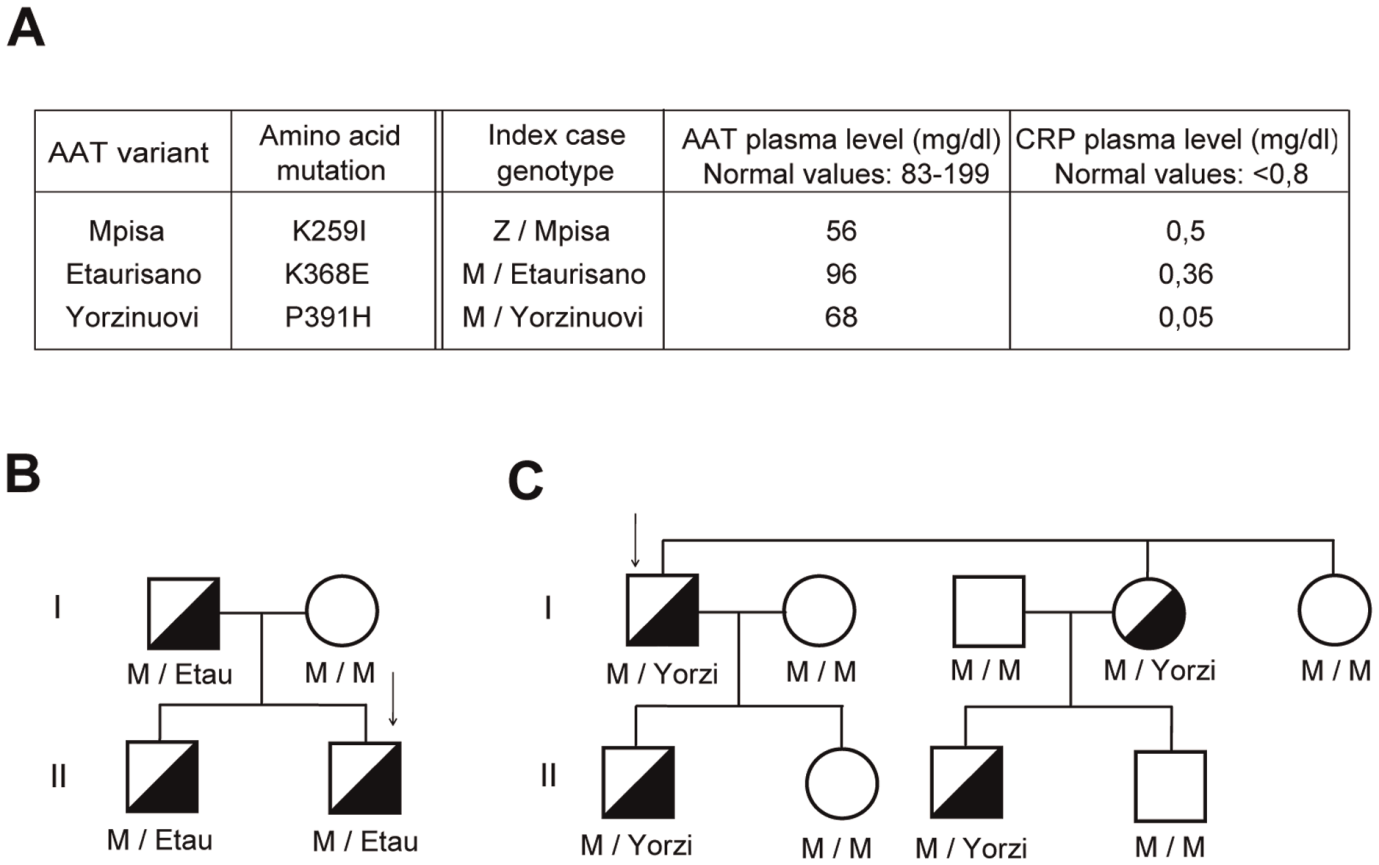
Genotypes of the index cases. (A) Table summarizing the amino acid mutations of the variants, the genotypes of the index cases as well as the plasma levels of AAT and C-reactive protein (CRP) determined in the index cases. (B) Pedigree of the Etaurisano proband’s family. (C) Pedigree of the Yorzinuovi proband’s family. Arrows in the family trees indicate index cases. Half-filled squares and circles indicate heterozygous subjects.

Here we report the identification and characterization of three novel AAT alleles, Mpisa (Lys259Ile), Etaurisano (Lys368Glu) and Yorzinuovi (Pro391His), associated with reduced concentration of AAT in plasma. The behaviour of the variants when expressed individually in cellular models clearly supports their identification as deficiency alleles, with a range of intracellular polymer loads observed. These mutations cluster structurally and are likely to facilitate polymerization both by effects upon ‘latch’ interactions constraining the AAT reactive loop and upon core packing.

## Materials and Methods

### Subjects

The index case carrying the Mpisa AAT allele was 26 years old at the time of diagnosis at the San Matteo Hospital (Pavia, Italy). He had dyspnea and no evidence of liver disease. The index case carrying the Etaurisano AAT allele was 31 years old when he was referred to the San Matteo Hospital (Pavia, Italy) because of panlobular emphysema. The proband with the Yorzinuovi allele was first admitted at the age of 46 at the Gastroenterology department of Spedali Civili (Brescia, Italy) because of a ten year history of mild hyper-transaminasemia. The index case’s families were investigated where possible. Collection of blood samples and the genetic investigations were performed after appropriate written informed consent of the subjects involved and approval by the ethical committees of the institutions involved (San Matteo Hospital of Pavia and Spedali Civili of Brescia, Italy). The patients in this manuscript have given written informed consent to publication of their anonymised case details.

**Figure 2:**
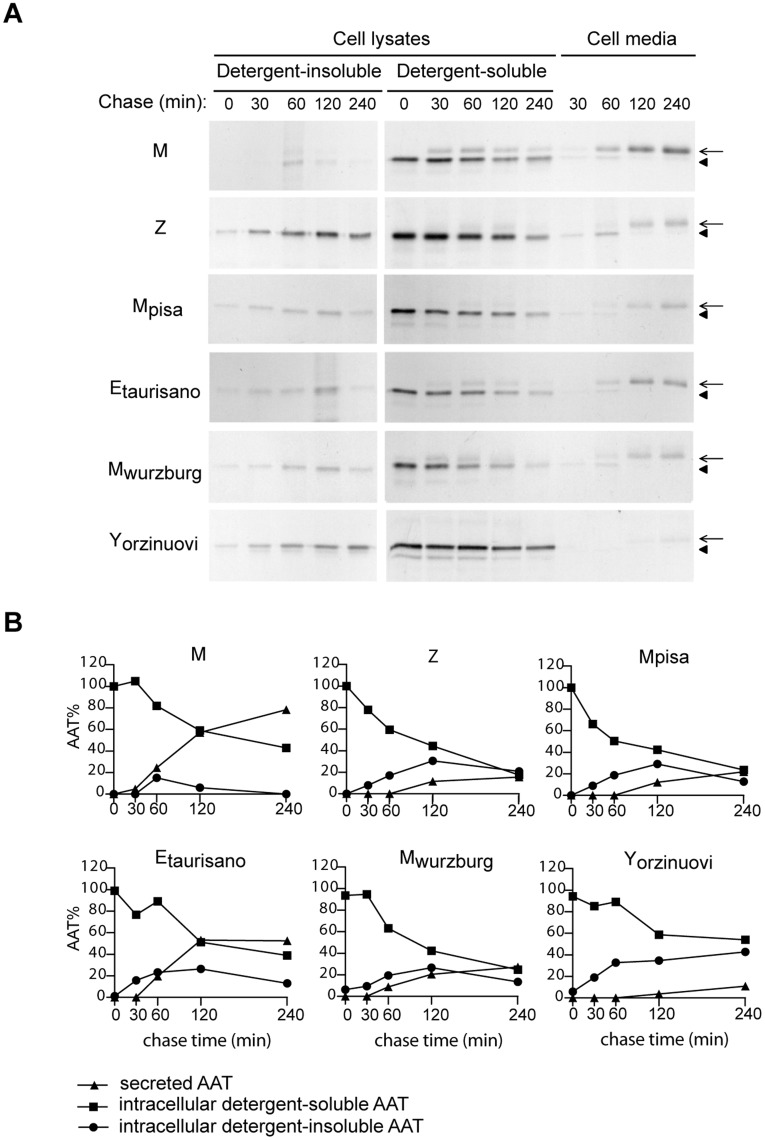
Fate of the new AAT **variants in Hepa 1.6 transfected cells.** (A) Pulse-chase experiments on Hepa 1.6 cells expressing AAT variants. Transfected Hepa 1.6 cells were pulsed with ^35^S Met/Cys for 10 min and chased for the indicated times. AAT was immunoprecipitated from culture media and from NP40-soluble and NP40–insoluble cell fractions, and analysed by SDS-PAGE and autoradiography. Arrows, mature secreted AAT (approx. 54 kDa); arrowheads, immature intracellular AAT (approx. 50 kDa). (B) Densitometric analysis of the autoradiograms shown in panel A. The relative amounts of AAT at the different times are expressed as percentage of the total amount of intracellular AAT at the beginning of chase (time 0), set as 100%.

### Quantification of Alpha1-antitrypsin and C-Reactive Protein (CRP) Plasma Levels

The AAT level measurements were performed on plasma or dried blood spot (DBS) samples by a rate immune nephelometric method (Array 360 System; Beckman-Coulter) using a goat anti-human AAT antibody (Beckman-Coulter) [21]. The CRP level measurement was performed on plasma samples by an immune nephelometric assay (Image Immunochemistry System; Beckman-Coulter).

**Figure 3:**
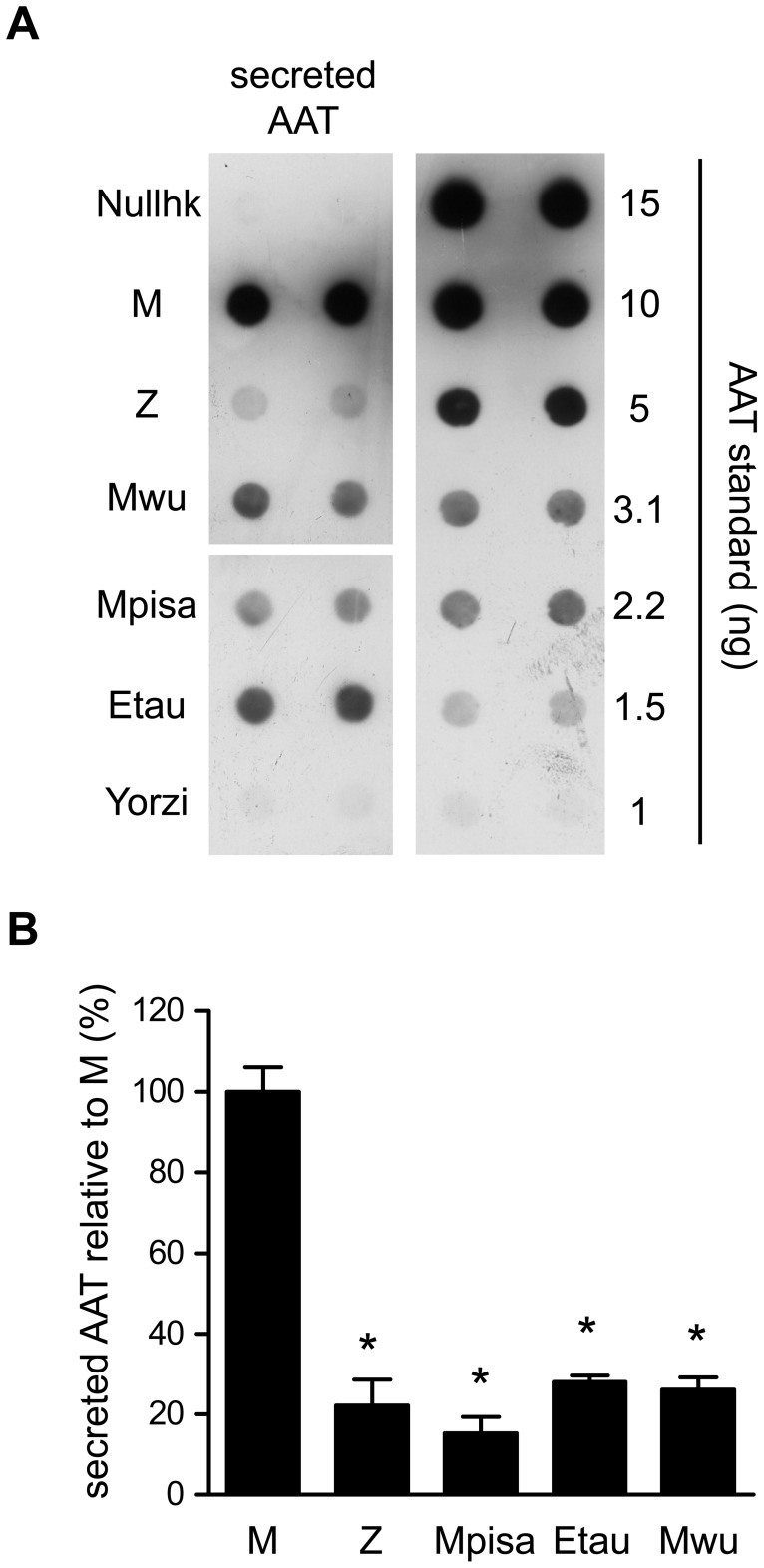
Secretion of the AAT variants relative to M AAT. (A) Dot blot analysis of AAT secreted in the cell media by Hepa 1.6 cells expressing Null-hk, M, Z, Mwurzburg, Mpisa, Etaurisano or Yorzinuovi. The right panel shows serial dilutions of purified AAT. (B) AAT secreted in the cell media of transfected Hepa1.6 cells, expressed as percentage of M AAT. Dot blots performed as shown in panel A were analysed by densitometry, AAT levels were calculated from the purified AAT standard curve, and expressed as percentages of M AAT level. The results are presented as means and SD of three independent transfection experiments (each experiment in duplicates). Asterisks indicate statistical significance (p<0.01 by Dunnett’s test of each variant compared to M AAT). Null-hk and Yorzinuovi were <10% of M AAT.

### Isoelectric Focusing (IEF) of Alpha1-antitrypsin

The phenotype of the new variants was determined by IEF analysis on plasma/DBS samples, comparing them with sera of known phenotype in a pH gradient of 4,2–4,9 (Multiphor II Electroforesis System, GE Healthcare BioScience) as previously described [22]. Conventionally, the variants were named according to their IEF migration and the birthplace of the senior carrier.

### Molecular Studies of New Alpha1-antitrypsin Variants

DNA was isolated from whole peripheral blood or DBS samples using a commercial extraction kit (DNA IQ System, Promega). Genotyping for detection of the S and Z variants was performed by *PCR/RFLP,* as previously described [23]. The new mutations were identified by sequencing all coding exons (II-V) of the AAT gene (SERPINA1, RefSeq: NG_008290), as previously described [22], using the CEQ 8800 genetic analysis System (Beckman Coulter). The background of the Mpisa and Yorzinuovi alleles was determined on the base of the rs709932, rs6647 and rs1303 SNPs. To determine the background of the Etaurisano variant, the amplimers of exon V from the index case’s DNA were subsequently cloned into a pCR2.1 vector using the TA Cloning Kit (Invitrogen), transformed in One Shot InvαF’ E.coli cells and sequenced by usual protocol.

### Construction of Alpha-1 Antitrypsin Expression Vectors

The pcDNA3.1/Zeo (+) expression vectors encoding for human M1(Val213) and Z AAT [24] were generous gifts from Dr R. Sifers (Baylor College of Medicine, Houston, TX). The mutations of the Mpisa, Etaurisano and Yorzinuovi were introduced into the M1 AAT cDNA by site-directed mutagenesis using the QuikChange II Site-Directed Mutagenesis Kit (Stratagene), according to the manufacturer’s instructions and with the following primers and those complementary to them:

Mpisa (5′CTGCCTGATGAGGGGATACTACAGCACCTG);

Etaurisano (5′CCCGAGGTCAAGTTCAACGAACCCTTTGTCTTCTTAA);

Yorzinuovi (5′TGGGAAAAGTGGTGAATCACACCCAAAAATAACTGCC).

### Cell Culture and DNA Transfections

The Hepa 1.6 (IZLER, Brescia, Italy) and COS-7 (ATCC) cells were grown in DMEM supplemented with 10% FBS (Sigma). Transient transfections of both cell lines with AAT encoding vectors were performed with Lipofectamine2000 (Invitrogen) according to the manufacturer’s protocol, using a 1∶3 DNA (µg)/Lipofectamine (µl) ratio in serum free DMEM for 5 h and then culturing transfected cells with normal medium for further 24 h. Alternatively, Hepa1.6 cells were transfected by adding to sub-confluent cells grown in normal culture medium a PBS solution containing polyethylenimine (PEI “Max”, Polysciences Inc.) and plasmid DNA (20 µg PEI “Max” and 2 µg DNA in 40 µl PBS for 10 cm^2^ wells).

### Pulse-chase Experiments

Cells were pulsed for 10 min with ^35^S Met/Cys (EasyTag™ Express Protein Labelling mix, Perkin Elmer) and chased for 0, 30, 60, 120 and 240 min. At each point in time, the cell media were collected and the cells were lysed in a buffer containing 50 mM Tris-HCl (pH 7.4), 150 mM NaCl, 1% NP40, 10 mM NEM and protease inhibitors (Sigma-Aldrich). The NP40-soluble and -insoluble fractions from cell lysates were separated by centrifugation at 12000 g and the insoluble material recovered in the pellet was solubilised by boiling in 50 mM Tris-HCl, pH 7.4, 2% SDS and then diluted 1∶10 with a buffer containing 50 mM Tris-HCl (pH 7.4), 150 mM NaCl, 1% NP40. The radiolabelled AAT in cell fractions and media was then immunoprecipitated using an anti-AAT polyclonal antibody (DAKO) and analysed by SDS-PAGE/autoradiography.

### Quantitative Dot Blot

The different AAT variants were expressed in Hepa 1.6 cells by transfection with Lipofectamine2000 (Invitrogen) as described above. 24 h after transfection, the culture media were replaced by serum-free media and cells were further incubated for 16 h. AAT in the cell media was analysed by dot blot in parallel with serial dilutions of purified AAT (Sigma), revealed with an anti-AAT polyclonal antibody (DAKO), HRP-conjugated anti-rabbit antibodies (Amersham) and ECL Plus (Amersham). In one experiment, AAT was revealed by anti-AAT polyclonal antibody (DAKO), IRDye800CW-conjugated anti-rabbit IgG (Li-cor Bioscience) and the Odissey FC Imaging System (Li-cor Bioscience). Dot blots were analysed by densitometry, and the AAT levels were calculated for each experiment from the AAT standard curve and expressed as percentages relatively to the mean levels for M AAT. Statistical analysis was performed by one-way ANOVA followed by Dunnet’s post-test using GraphPad Prism version 5 (Graphpad software Inc, San Diego, CA, USA).

### Non-denaturing PAGE and Western Blot Analysis

For non-denaturing analysis, the different AAT variants were expressed in COS-7 cells by transfection with Lipofectamine2000 (Invitrogen) as previously described [25]. 48 h after transfection culture media were collected and concentrated 10 times in Vivaspin columns with a 3000 Da cut-off membrane (VivaScience), while cells were lysed in a NP40 buffer (150 mM NaCl, 50 mM Tris-HCl pH 7.4, 1% NP40). Both cell lysates and culture media were subjected to 7.5% non-denaturing PAGE and blotted as described [25]. Membranes were probed with either the commercial anti-AAT mAb 704 (Abcam) or with the 2C1 mAb [18] and revealed with HRP-conjugated anti-mouse antibodies (Sigma-Aldrich) and ECL SuperSignal West Pico and Femto Maximum Sensitivity Substrates (Pierce Biotechnology).

## Results

### Identification of the Novel Alpha1-antitrypsin Alleles

The new AAT variants investigated in this study, as well as the genotype and plasma concentration of AAT and CRP of the index cases, are summarised in Figure 1A. The levels of CRP were determined in order to exclude the influence of an acute inflammatory state on AAT plasma concentration. The variants were named according to the plasma protein’s migration by IEF (M, E and Y; Figure S1) and the birthplace of the senior carrier (Pisa, Taurisano and Orzinuovi).

The Mpisa allele is characterized by an A to T transition in exon III (c.848A>T) in the M1Val background, resulting in a Lys259Ile substitution. The Mpisa allele was identified in association with the Z AAT allele in a patient with emphysema and reduced AAT plasma levels (56 mg/dL). Afterwards, other five M/Mpisa unrelated subjects were identified. Their AAT plasma levels (82–96 mg/dl) were at the lower limit of normal values [26].

The Etaurisano allele is characterized by a transversion in AAT exon V (c.1174 A>G) resulting in Lys368Glu substitution. It was first identified in a subject with panlobular emphysema, in the heterozygous state with the M allele. It was then found in the index case’s father and brother (Figure 1B). Cloning and sequencing of exon V indicated that the Etaurisano mutation arose on the M2 background allele.

The Yorzinuovi allele is characterised by a transversion in exon V (c.1244C>A), resulting in a Pro391His protein mutation, on a M1Val background. It was first identified in heterozygous state with M in a subject with mild but persistently elevated transaminase levels, as well as in three related asymptomatic subjects (Figure 1C). Notably, the Pro391His mutation was previously found in association with the Asp256Val mutation in the Ybarcelona AAT variant [27].

More clinical details on the probands are reported in the Information S1.

**Figure 4:**
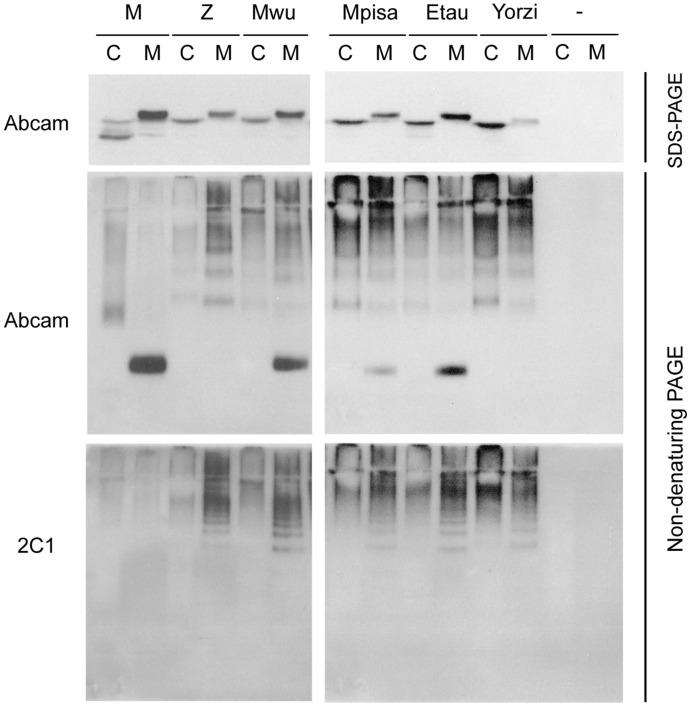
Intracellular accumulation of the new AAT variants as 2C1-positive polymers. PAGE and immunoblot analysis for AAT in NP40–soluble cellular fractions (C) and culture media (M) from COS-7 cells transfected with M, Z, Mwurzburg, Mpisa, Etaurisano and Yorzinuovi AAT cDNAs or empty vector (-). Top panels: reducing SDS-PAGE and immunoblot with the 704 mAb to AAT (Abcam). Middle panels: non-denaturing PAGE and immunoblot with the 704 mAb (Abcam). Bottom panels: non-denaturing PAGE and immunoblot with the 2C1 mAb specific for AAT polymers. The middle and bottom panels correspond to the same membranes probed first with the 2C1 mAb and then with the 704 mAb from Abcam.

### Reduced Secretion of the Newly Identified Variants by Hepa 1.6 Hepatoma Cells

We investigated the molecular behaviour of the novel AATD-associated variants by expressing them in liver-derived cells (Hepa1.6) and performing pulse-chase experiments (Figure 2). As controls we included M and Z AAT, as well as Mwurzburg (Pro369Ser), a previously described AAT deficiency variant whose amino acid substitution occurs next to that of Etaurisano [28]. As expected, the newly synthesized M AAT was present at the end of the pulse period in the NP40-soluble cell fraction as a single band of about 50 kD, which corresponds to the high mannose intermediate form (Figure 2A, arrowhead). During the chase period, the protein is converted to a fully glycosylated mature protein (approx. 54 kD) that is rapidly secreted into the medium (Figure 2A, arrow). Only a small amount of M AAT was transiently found as immature polypeptide in the NP40-insoluble cell fraction. On the other hand, the newly synthesized Z protein was mainly retained in the cells as immature and significantly accumulated in the NP40-insoluble fractions, confirming its tendency to form large insoluble complexes (Figure 2A). The novel variants showed a reduction of protein secretion and were found in the NP40-insoluble fraction, suggesting that they formed large insoluble intracellular complexes similarly to Z AAT, although to different extents. The AAT distribution at different time points in the intracellular soluble and insoluble fractions, as well as in the cell media, was quantified by densitometry and expressed as percentages of the total AAT amount found at time 0 of chase (Figure 2B). At 240 min of chase, when about 78% of the wild-type M is found in the culture medium, the different mutants show reduced secretion (Z 16%, Mwurzburg 27%, Etaurisano 52%, Mpisa 22% and Yorzinuovi 11%). At the same time point, when the wild-type M is undetectable in the NP40-insoluble fraction, all the mutants show an accumulation of detergent insoluble complexes (Z 21%, Mwurzburg 13%, Etaurisano 13%, Mpisa 13% and Yorzinuovi 43%).

The secretion efficiency of the variants was also evaluated at steady state by quantitative dot-blot analysis of the AAT found in cell culture media of transfected Hepa1.6 cells (Figure 3). Panel A represents a single experiment comparing the different mutant variants and the M, Z and Null-hongkong (Null-hk) controls, using serial dilutions of commercial purified AAT (Sigma) as reference. Quantitative densitometric analysis of dot blots from three independent transfection experiments (Figure 3B) confirmed the results of the pulse chase experiments showing that the new deficiency variants all behave as defective in Hepa 1.6 cells, the Yorzinuovi being the most severely deficient (below 10% of M AAT).

### Formation of Polymers by the Alpha1-antitrypsin Variants in Transfected COS-7 Cells

The finding that the new variants accumulated as large NP40-insoluble complexes in Hepa 1.6 cells prompted us to investigate whether their accumulation occurs in the form of ordered polymers, similarly to Z AAT. We assessed the presence of AAT polymers in cell lysates and culture media from COS-7 cells transiently expressing the variants by non-denaturing PAGE and immunoblot analysis (Figure 4). As expected, M AAT was found at low levels within the cells and was secreted in the cell medium exclusively in the monomeric form. Z AAT instead was found mainly as polymers both in the cell lysate and in the medium. Similarly, polymeric AAT was found in both the cell lysates and media of the cells expressing the new variants (Figure 4, middle panels). Variable proportions of the monomer and polymer forms are detected for the different AAT variants, in correlation with their propensity to polymerize. This is consistent with previous studies on alpha1-antitrypsin and neuroserpin mutants [18], [25], [29]. The Mwurzburg and Etaurisano variants displayed a higher percentage in the monomeric form, in agreement with their milder defect in secretion. Based on our results, we cannot determine whether the polymers found in the cell media are formed extracellularly, secreted from the cells or both. The polymeric forms were all recognised by the polymer specific mAb 2C1 [18] (Figure 4, bottom panels), suggesting that the polymers formed by the new variants of AAT structurally resemble those formed by Z AAT. Analysis of intracellular and extracellular AAT variants by sandwich ELISA with the 2C1 mAb [30] confirmed the presence of Z-like polymers in all the samples produced by transient expression of the new AAT variants in COS-7 cells (results not shown).

### Structural Modelling of the New Mutant Variants of AAT

**Figure 5:**
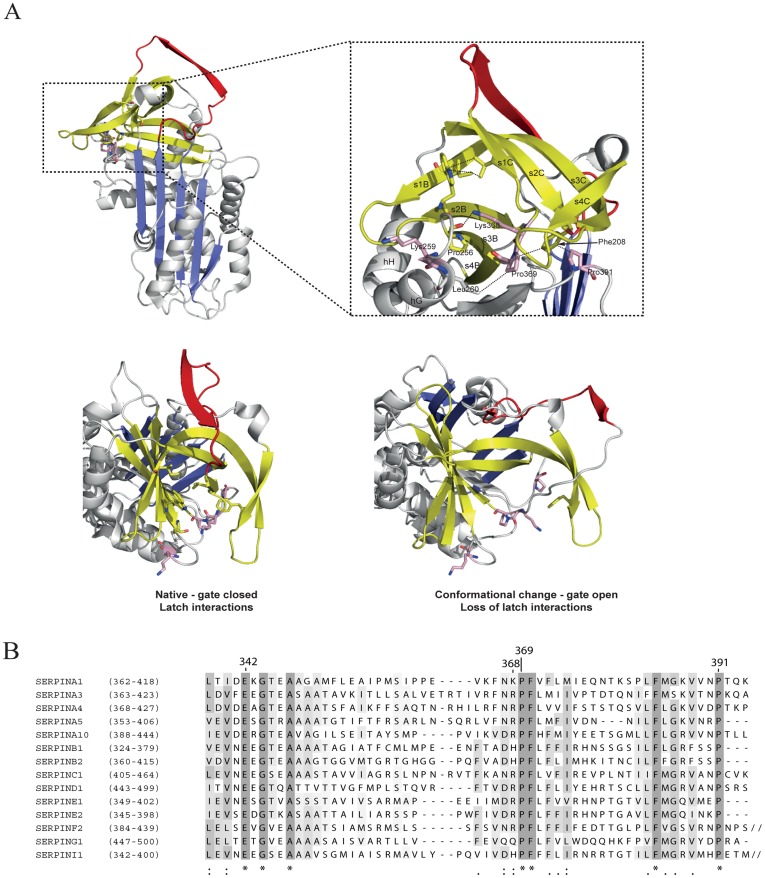
Structural analysis of the new alpha1-antitrypsin mutations. (A) Upper panel: Mutation sites in native AAT (PDB ID: 1QLP; β-sheet A in blue, reactive loop red, B/C sheet β-barrel yellow, mutation site residues in pink, zoom is rotated 90° around vertical axis). ‘Latch’ interactions (dotted lines) stabilise the closed conformation of the ‘gate’ (s4C-s3C). Lower panel: left, gate closure in the native state; right, gate opening is associated with loss of latch interactions, release of s1C and conformational change (intermediate state modelled upon latent conformer, PDB ID: 1IZ2, [39]). (B) Alignment of C-terminal sequences of inhibitory human serpins, performed by ClustalW2 (www.ebi.ac.uk, default parameters, visualised by Jalview). SERPINA1, α_1_-antitrypsin; A3, α_1_-antichymotrypsin; A4, kallistatin; A5, PAI3; A10, protein Z-dependent protease inhibitor; B1, leukocyte elastase inhibitor; B2, PAI2; C1, antithrombin III; D1, heparin cofactor 2; E1, PAI1; E2, glia-derived nexin; F2, α_2_-antiplasmin; G1, C1 inhibitor; I1, neuroserpin. Alpha1-antitrypsin mutation sites of Z (342), Etaurisano (368), Mwurzburg (369) and Yorzinuovi (391) are indicated (top).

We have assessed likely structural effects of the new mutations (Figure 5). They cluster in the so-called ‘gate’ region of AAT [31], where a network of hydrophobic interactions fasten the lateral border of β-sheet C to close a β-barrel motif with β-sheet B. The Yorzinuovi, Etaurisano and Mwurzburg mutations loosen this interaction to facilitate release/prevent tethering of strand 1 of β-sheet C (s1C) and favour mobilisation of the reactive site loop to adopt alternative conformations (Figure 5A). The Mpisa (Lys259Ile), Etaurisano (Lys368Glu) and Mwurzburg (Pro369Ser) mutations will also perturb helix G, affecting core packing and hence the shutter region.

AAT is the archetype of the serine protease inhibitor (serpin) superfamily [32]. We performed protein sequence alignment of a range of inhibitory serpins [33] to assess the overall conservation of the aminoacids mutated in the new variants. The Yorzinuovi (Pro391His), Etaurisano (Lys368Glu) and Mwurzburg (Pro369Ser) mutations affect highly conserved C-terminal residues (Figure 5B), further supporting the hypothesis that they play a crucial role in the folding, stability and function of serpins.

## Discussion

In this study we report three new AAT deficiency variants, bearing non-conservative aminoacid substitutions, that we named Mpisa (Lys259Ile), Etaurisano (Lys368Glu), and Yorzinuovi (Pro391His). These variants were identified in Italian patients with reduced serum AAT levels. The previously reported variant Mwurzburg was included in our study because the causative mutation (Pro369Ser) affects the adjacent residue to that affected in Etaurisano (Lys368Glu). Moreover, this variant is represented in the Italian cohort of AATD patients with rare alleles (LC and ML, unpublished data) and allows us a further correlation between mutations in this region and cell model data. We studied both intracellular accumulation and defective secretion of AAT in our cell models since these respectively relate to the major pathogenic drivers of the liver and lung disease associated with AAT deficiency.

The new variants show a secretion defect in Hepa cells, the Yorzinuovi being the most severely deficient. These in vitro results are fully consistent with the plasma levels observed in the carriers, while clinical evidence of lung disease was established only in some patients. The onset of emphysema indeed depends on several risk factors, particularly cigarette smoke, as widely documented for Z homozygous individuals [6].

We also evaluated the tendency of the different variants to form ordered polymers when expressed in transfected Hepa and COS cells. The higher tendency to form polymers was displayed by Yorzinuovi, which explains its severe secretory defect. Based on the recognition by the 2C1 polymer-specific monoclonal antibody, the structure of the polymers formed by the new variants resembles that of Z. The index case carrying the Yorzinuovi allele in association with the wild-type M allele was referred because of persistently elevated transaminases, although no AAT inclusions were evidenced by immunohistochemical analysis on liver biopsy, while the two other carriers of this allele were fully asymptomatic. This mild phenotype may be related to the heterozygote state of the carriers. Similarly the MZ phenotype is not associated with severe lung or liver disease. Indeed, even the ZZ phenotype is associated with a wide spectrum of liver disease: from disease-free to end-stage hepatic failure or malignancy. Thus the risk of liver disease from intrahepatocytic polymerization is modified by further genetic and environmental factors, only partially elucidated.

Sequence alignment of different serpins revealed that the aminoacids mutated in the Etaurisano, Mwurzburg and Yorzinuovi are in a highly conserved C-terminal region, likely to be important for the structural stability of serpins. Consistently, other natural mutations leading to plasma deficiency have been reported to affect this region. The Pro391His mutation was previously found in association with the Asp256Val mutation in the Ybarcelona AAT variant [27]. Pro369 is also mutated in the Mheerlen AAT deficient variant (Pro369Leu) [28], [34]. Moreover, the Pro476Ser substitution in the C1 inhibitor (equivalent to Pro391 in the AAT) was previously reported to promote intracellular multimerization, causing reduced plasma levels and angioedema [35].

The crucial role of the C-terminal AAT region is strengthened by our structural analysis aimed at predicting the possible effects of the mutations underlying intracellular accumulation and polymer formation. The mutations highlight the structural significance of the gate region of AAT, where a network of hydrophobic interactions fasten the lateral border of β-sheet C to close a β-barrel motif with β-sheet B. Since these interactions keep the gate region closed in the native structure we propose they be termed ‘latch’ interactions. Loosening these interactions will favour ‘unlatching’ of the gate and population of the polymerogenic intermediate M* (Figure 5A). An opened gate [31] and untethered s1C [36] have both previously been proposed as requirements for conformational change in the serpin superfamily. The new mutations show these are likely intrinsically coupled during intracellular polymerisation. The network of latch interactions also explains the elegant systematic mutagenesis study of Pro391 carried out almost two decades ago [37]. Mutation to hydrophobic residues had minimal effects on secretion and degradation profiles. However as mutations became increasingly hydrophilic, intracellular retention within the ER and/or degradation increased to give phenotypes more marked than the Z variant.

The mutations also highlight the significance of packing interactions between α-helices A and G and β-sheet B within the core of the molecule. Destabilisation of such interactions and consequent dysregulation of β-sheet A opening has been postulated as the mechanism by which the S (Glu264Val) [38] and I (Arg39Cys) [20] mutations cause polymerisation and mild plasma deficiency. The Mpisa (Lys259Ile) mutation will affect G-helix packing whilst the linker preceding the G-helix will be destabilised by the Etaurisano (Lys368Glu) mutation. Formation of the intermediate state in either the single strand (‘classical’ loop-sheet model [12], [13]) or triple strand (C-terminal derived [15]) linkage models of serpin polymerization are highly consistent with direct coupling of s1C release with intramolecular loop insertion. Both of these models are therefore similarly compatible with the finding that gate loosening and/or core disrupting mutations result in increased polymerization and mild deficiency. In contrast, gate opening does not appear integral to the 2 strand (beta-hairpin [14]) model. It seems less likely that the ‘latch’ mutations we describe would trigger polymer formation by this mechanism. Further structural studies will be required to establish whether the novel variants populate extensively unfolded intermediates in the disease state, as proposed in the triple-strand model, or favour more parsimonious conformational change, consistent with single-strand linkage. In conclusion, we demonstrate that the new AAT deficiency variants, besides increasing the risk of lung disease, may predispose to liver disease, particularly if associated with Z or with a different polymerogenic variant. Together the novel mutations define a latch region that regulates the population of an M* state. Polymerogenic mutations here will promote conformational change by favouring s1C release and/or effects upon core packing.

## Supporting Information

Figure S1 **Phenotyping of the new AAT variants.** IEF analysis was performed on plasma from the heterozygous carriers of the new AAT variants, comparing them with plasma of known phenotype in a pH gradient of 4,2–4,9. The arrow shows the major band of the Etaurisano variant. The asterisk shows the major band of Yorzinuovi.
(TIF)

Information S1 **Clinical data.** Supplementary clinical information on the index cases carrying the Mpisa, Etaurisano and Yorzinuovi AAT alleles.
(DOC)
