# Supplementary figures and images for: Three New Alpha1-Antitrypsin Deficiency Variants Help to Define a C-Terminal Region Regulating Conformational Change and Polymerization
Source: PLoS One. 2012 Jun 18;7(6):e38405. doi: 10.1371/journal.pone.0038405 (PMC3377647; doi:10.1371/journal.pone.0038405)

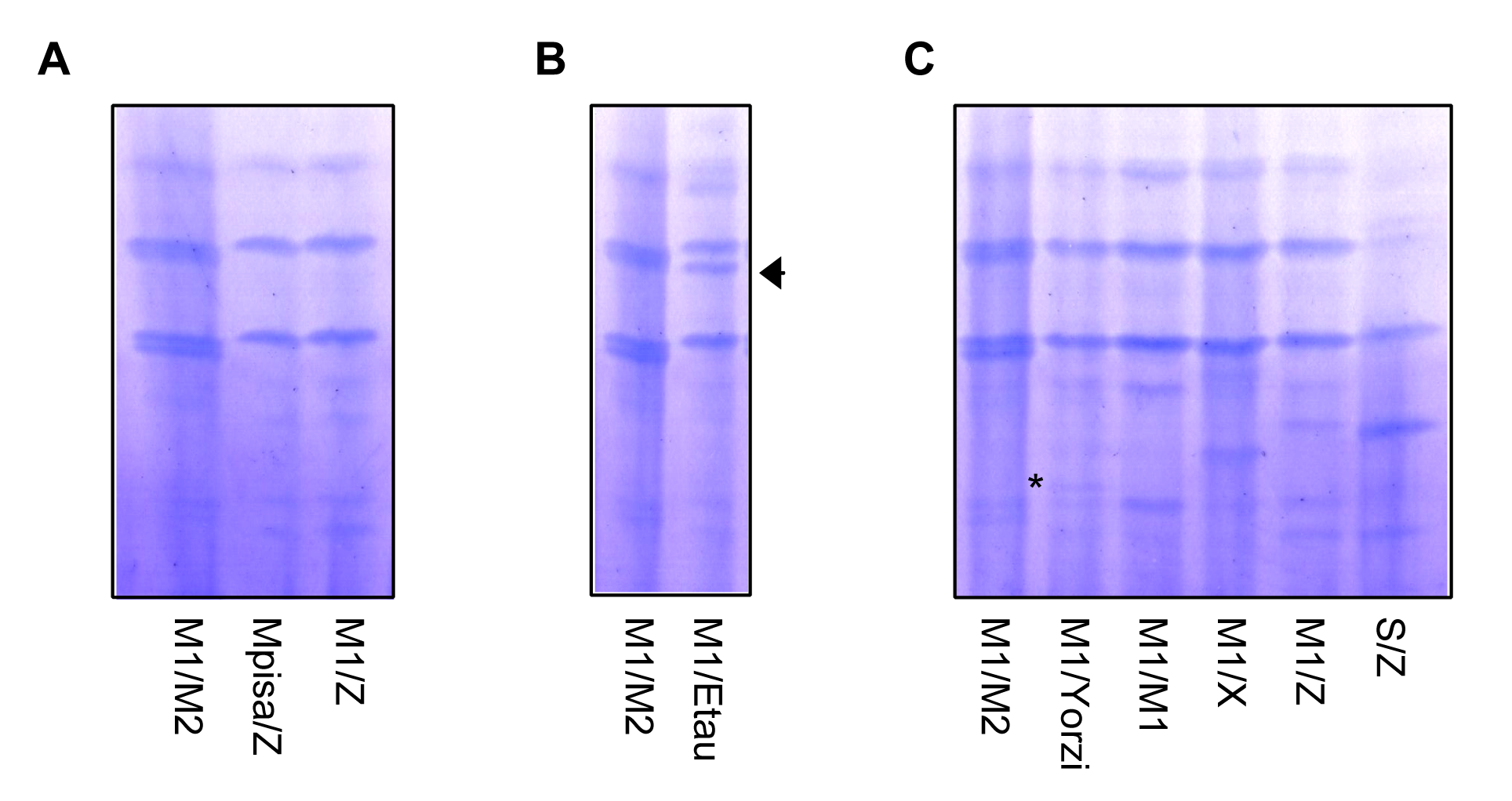

Supplement: Figure S1 — Phenotyping of the new AAT variants. IEF analysis was performed on plasma from the heterozygous carriers of the new AAT variants, comparing them with plasma of known phenotype in a pH gradient of 4,2–4,9. The arrow shows the major band of the Etaurisano variant. The asterisk shows the major band of Yorzinuovi. (TIF) [file pone.0038405.s001.tif]
