# Supplementary material for: Three New Alpha1-Antitrypsin Deficiency Variants Help to Define a C-Terminal Region Regulating Conformational Change and Polymerization
Source: PLoS One. 2012 Jun 18;7(6):e38405. doi: 10.1371/journal.pone.0038405 (PMC3377647; doi:10.1371/journal.pone.0038405)
Supplement: Information S1 — Clinical data. Supplementary clinical information on the index cases carrying the Mpisa, Etaurisano and Yorzinuovi AAT alleles. (DOC) [file pone.0038405.s002.doc]

**Clinical data**

The index case carrying the Mpisa AAT allele was 26 years old at the time of diagnosis at the San Matteo Hospital in Pavia. He had dyspnea, no smoking history and no evidence of liver disease. The AAT and CRP plasma levels determination showed values of 55.9 mg/dl and 0.5 mg/dl, respectively. No family study could be performed for this patient.

The index case carrying the Etaurisano AAT allele was 31 years old at the time of diagnosis. He was referred to the San Matteo Hospital in Pavia because of panlobular emphysema mainly in the lower pulmonary lobes and the lung function test showed FEV_1_ 1.40 L (33% predicted), FVC 2.88 L (57% predicted), VC 2.96 L (56% predicted) prebronchodilatator, and FEV1 2.60 L (61% predicted) , FVC 4.40 L (88% predicted) and VC 4.87 L (93% predicted) post-bronchodilatator. The arterial blood gas analysis showed a saturation of 96%. Liver function tests were within the normal range. The AAT and CRP plasma levels were 96,2 mg/dl and 0.36 mg/dl respectively. He smoked 17.5 pack/year. The index case’s father and brother, both carrying the Etaurisano allele, had no evidence of lung and liver disease, and AAT plasma levels of 119,1 mg/dl and 135.6 mg/dl, respectively. At the time of the diagnosis they were ex-smokers (0.6 and 0.2 and pack/year, respectively).

The index case carrying the Yorzinuovi AAT allele was admitted at the age of 46 to the Gastroenterology department (Spedali Civili, Brescia, Italy) because of a ten year history of mild asymptomatic hyper-transaminasemia. On admission the patient reported a familiar history of HFE-related haemochromatosis (his father died for liver cirrhosis). The patient denied alcohol and drug consumption, and his medical history was unremarkable; his physical examination was normal except that a mild palpable hepatomegaly and body overweight (body mass index = 27).

Laboratory tests confirmed increased serum levels of alanine aminotransferase (108 IU/L; normal value < 50 IU/L) and of aspartate aminotransferase (75 IU/L; normal value < 35 IU/L), and revealed hypercolesterolemia (236 mg/dl, normal value < 200 mg/dl) and hyperferritinemia (342 ng/ml, normal value < 300), with normal values of transferring saturation rate (35%). Genetic analysis of HFE revealed a singleH63D mutation. No other abnormal findings were found at serological blood tests - including virological, metabolic and autoimmune serological examinations - except decreased AAT serum levels (59 mg/dL). Sequence analysis of the *SERPINA1* gene revealed the presence of the normal M allele in association with the novel Yorzinuovi allele. Lung functional tests were found normal. Ultrasonography of the abdomen revealed fatty liver pattern without any sign of liver fibrosis. Histological analysis of a liver biopsy revealed an inflammatory pattern, mild steatosis but was negative for PAS+, diastase resistant inclusions and for AAT accumulation. Three other heterozygous asyntomatic carriers of the Yorzinuovi were found in the family study, showing AAT plasma values of 68, 87 and 68 mg/dL, respectively.
